# Supplementary material for: Analysis of PPARγ Signaling Activity in Psoriasis
Source: Int J Mol Sci. 2021 Aug 10;22(16):8603. doi: 10.3390/ijms22168603 (PMC8395241; doi:10.3390/ijms22168603)
Supplement: Supplementary file 1 [file ijms-22-08603-s001.zip › Supplemental materials_Analysis of PPARg signaling activity in psoriasis/Pathway models/Models images and html files/Model of PPARG related pathways in psoriasis (short version)/6369.html]

IL12A


# Protein IL12A

|  |  |
| --- | --- |
| URN | urn:agi-llid:3592 |
| Total Entities | 0 |
| Connectivity | 872 |
| Name | IL12A |
| Description | interleukin 12A |
| Notes | This gene encodes a subunit of a cytokine that acts on T and natural killer cells, and has a broad array of biological activities. The cytokine is a disulfide-linked heterodimer composed of the 35-kD subunit encoded by this gene, and a 40-kD subunit that is a member of the cytokine receptor family. This cytokine is required for the T-cell-independent induction of interferon (IFN)-gamma, and is important for the differentiation of both Th1 and Th2 cells. The responses of lymphocytes to this cytokine are mediated by the activator of transcription protein STAT4. Nitric oxide synthase 2A (NOS2A/NOS2) is found to be required for the signaling process of this cytokine in innate immunity. [provided by RefSeq, Jul 2008] |
| Primary Cell Localization | Extracellular |
| Class | Ligand |

---

|  |  |
| --- | --- |
| Pathway | Toll-like Receptors Act through MYD88-TIRAP Signaling |
|  | Dectin-1 (CLEC7A) Signaling |
|  | DCIR1 (CLEC4A) Signaling |
|  | TLR4 Signaling in Leukocytes |
|  | CD8+ T-Cell Activation |
|  | Th1-Cell Differentiation |
|  | Toll-like Receptors in Sterile Inflammation |
|  | Lipids Enhance Apoptotic Cell Engulfment and Reduce Inflammation |
|  | Macrophage M1 Lineage |
|  | AXL Receptor Inhibits Macrophages and Dendritic Cells Function |
|  | Lymphocyte Mediated Myocardial Injury in Myocarditis |
|  | Inflammatory Reaction in Acne Vulgaris |
|  | Atopic Dermatitis |
|  | Dendritic Cells Function in Psoriasis |
|  | Apoptotic Keratinocytes Clearance Recession in Systemic Lupus Erythematosus |
|  | Th1-Cell Function in Systemic Lupus Erythematosus |
|  | T-Cell Cytotoxic Response against Melanocytes in Vitiligo |
|  | TLR7 and TLR9 in Plasmacytoid Dendritic Cell |
|  | CD4+T-Cell Response in Celiac Disease |
|  | Crohn's Disease |
|  | Th1-Cell Activation in Crohn's Disease |
|  | Th17-Cell Activation in Crohn's Disease |
|  | Dendritic Cell Function in Ulcerative Colitis |
|  | Il17 Signaling Related Neutrophilia in Asthma |
|  | Dendritic Cell Dysfunction in Psoriatic Arthritis |
|  | IL6/IL12 Signaling Activates Immune System in Multiple Sclerosis |
|  | Immune System Role in Otitis Media |
|  | Proteins Involved in Chronic Obstructive Pulmonary Disease |
|  | STAT3 Inhibits Maturation of Dendritic Cells and Facilitates Cancer Progression |
|  | Myeloid Derived Suppressor Cells in Cancer Immune Escape |
|  | EGFR -> Expression Targets in Skin |
|  | IFNG/IFNR Expression Targets |
|  | IL23R -> STAT3/NF-kB Signaling |
|  | C5AR1 -> Expression Targets in Lymphoid System and Blood |
|  | TLR3 Expression Targets |
|  | TLR4 -> AP-1 Expression Targets |
|  | TLR4 -> NF-kB/IRF Expression Targets |
|  | Model of PPARG related pathways in psoriasis (short version) |
|  | 1\_Inflammation in keratinocytes in the pathology of acne |
|  | 1\_Defects in response to pathogens in the gut promotes inflammation in Crohn's disease |
|  | 2\_Intestine epithelial cells drive immunological responses in ulcerative colitis |
|  | 1\_Antigen-presenting cells promote islet dysfunction and immune system activation in T1D |
|  | 2\_Defective tolerance of autoreactive T-cell in T1D |

---

|  |  |
| --- | --- |
| MedScan ID | 3592 |

---

|  |  |
| --- | --- |
| LocusLink ID | 3592 |
|  | 16159 |
|  | 84405 |

---

|  |  |
| --- | --- |
| Alias | MGC151232 |
|  | cytotoxiclymphocyte maturating factor 1, p35 |
|  | cytotoxic lymphocyte maturation factor I |
|  | natural killer cell stimulatory factor 1 |
|  | Interleukin-12 alpha |
|  | interleukin IL-12, subunit p35 |
|  | cytotoxiclymphocyte maturation factor 1, p35 |
|  | NF cell stimulatory factor chain I |
|  | CLMF P35 |
|  | NKSF1 |
|  | Ll12a |
|  | Interleukin IL12A |
|  | NK cell stimulatory factor chain I |
|  | IL-12p35 |
|  | Cytotoxic lymphocyte maturating factor 35 kDa subunit |
|  | interleukin 12 p35 subunit |
|  | interleukin 12A |
|  | Interleukin IL12 alpha |
|  | natural killer cell stimulatory factor 1, 35 kD subunit |
|  | IL-12-specific subunit p35 |
|  | interleukin 12a p35 subunit |
|  | IL-12 p35 subunit |
|  | cytotoxic lymphocyte maturation factor 1 |
|  | MGC151228 |
|  | cytotoxic lymphocyte maturation factor 1, p |
|  | interleukin 12 alpha subunit |
|  | interleukin 12 subunit p35 |
|  | interleukin 12A (natural killer cell stimulatory factor 1, cytotoxic lympho |
|  | cytotoxic lymphocyte maturation factor 1, p35 |
|  | OTTHUMP00000213704 |
|  | cytotoxic lymphocyte maturating factor 1 |
|  | natural killer cell stimulatory factor I |
|  | interleukin IL-12p35 |
|  | NF cell stimulatory factor chain 1 |
|  | I12A\_HUMAN |
|  | interleukin-12 subunit alpha |
|  | IL-12, subunit p35 |
|  | cytotoxic lymphocyte maturating factor 1, p35 |
|  | NFSK |
|  | interleukin IL-12-specific subunit p35 |
|  | cytotoxic lymphocyte maturating factor I |
|  | CLMF |
|  | NK cell stimulatory factor chain 1 |
|  | interleukin 12A (natural killer cell stimulatory factor 1, cytotoxic lymphocyte maturation factor 1, p35) |
|  | Cytotoxic lymphocyte maturation factor 35 kDa subunit |
|  | NKSF I |
|  | OTTHUMP00000213705 |
|  | interleukin 12, p |
|  | IL12A |
|  | interleukin 12, p35 |
|  | P35 |
|  | IL-12A |
|  | IL35 subunit |
|  | interleukin-12 alpha chain |
|  | IL-12 subunit p35 |
|  | interleukin-12 p35 subunit |

---

|  |  |
| --- | --- |
| GO ID | 0005125 |
|  | 0008083 |
|  | 0042163 |
|  | 0005143 |
|  | 0045513 |
|  | 0046982 |
|  | 0035711 |
|  | 0035744 |
|  | 0007050 |
|  | 0016477 |
|  | 0008283 |
|  | 0071222 |
|  | 0098586 |
|  | 0019221 |
|  | 0050830 |
|  | 0042832 |
|  | 0097191 |
|  | 0006955 |
|  | 0035722 |
|  | 0070757 |
|  | 0032700 |
|  | 0048662 |
|  | 0051135 |
|  | 0045582 |
|  | 0001916 |
|  | 0042102 |
|  | 0045785 |
|  | 2000510 |
|  | 0032729 |
|  | 0050671 |
|  | 0032946 |
|  | 0032816 |
|  | 0045954 |
|  | 0002860 |
|  | 0034393 |
|  | 0042531 |
|  | 0010224 |
|  | 0032496 |
|  | 0009615 |
|  | 0009986 |
|  | 0005788 |
|  | 0005576 |
|  | 0005615 |
|  | 0043514 |
|  | 0031906 |
|  | 0005737 |
|  | 0032609 |
|  | 0010469 |
|  | 0042520 |

---

|  |  |
| --- | --- |
| KEGG ID | mmu:16159 |
|  | rno:84405 |
|  | hsa:3592 |

---

|  |  |
| --- | --- |
| Organism | Homo sapiens {Organism urn:agi-taxid:9606} |
|  | Mus musculus {Organism urn:agi-taxid:10090} |
|  | Rattus norvegicus {Organism urn:agi-taxid:10116} |
|  | Homo sapiens |
|  | Mus musculus |
|  | Rattus norvegicus |

---

|  |  |
| --- | --- |
| Mouse chromosome position | 3 31.92 cM |
|  | 3 37.0 cM |

---

|  |  |
| --- | --- |
| OMIM ID | 161560 |

---

|  |  |
| --- | --- |
| Rat chromosome position | 2q32 |
|  | 2q31 |

---

|  |  |
| --- | --- |
| Hugo ID | 5969 |
|  | HGNC:5969 |

---

|  |  |
| --- | --- |
| Human chromosome position | 3q25.33 |
|  | 3q25.33-q26 |

---

|  |  |
| --- | --- |
| Swiss-Prot Accession | O60595 |
|  | P29459 |
|  | P29459.2 |
|  | F8WI71 |
|  | P43431 |
|  | Q549G3 |
|  | P43431.1 |
|  | Q9R103 |
|  | Q9R103.1 |
|  | Q96QZ1 |
|  | E9PGR3 |
|  | Q7LDP2 |
|  | Q9QUT1 |
|  | E9QP58 |

---

|  |  |
| --- | --- |
| PIR ID | I56135 |
|  | B38957 |

---

|  |  |
| --- | --- |
| GenBank ID | NC\_000003 |
|  | NM\_000882 |
|  | NP\_000873 |
|  | NM\_001354582 |
|  | NP\_001341511 |
|  | NM\_001354583 |
|  | NP\_001341512 |
|  | NG\_033022 |
|  | AC010370 |
|  | AF050083 |
|  | AAC05211 |
|  | AAC05212 |
|  | AF404773 |
|  | AAK84425 |
|  | CH471052 |
|  | EAW78656 |
|  | EAW78657 |
|  | EAW78658 |
|  | CS498405 |
|  | CAM82732 |
|  | HH821934 |
|  | CBX32667 |
|  | JA104599 |
|  | CCA63969 |
|  | AF101062 |
|  | AAD16432 |
|  | AF180562 |
|  | AAD56385 |
|  | AW472810 |
|  | BC035571 |
|  | BC104982 |
|  | AAI04983 |
|  | BC104984 |
|  | AAI04985 |
|  | BG702253 |
|  | M65271 |
|  | AAA35694 |
|  | M65291 |
|  | AAA59937 |
|  | P29459 |
|  | NC\_000069 |
|  | NM\_001159424 |
|  | NP\_001152896 |
|  | NM\_008351 |
|  | NP\_032377 |
|  | XM\_006501044 |
|  | XP\_006501107 |
|  | AC121306 |
|  | AH004858 |
|  | AAB37382 |
|  | CH466547 |
|  | EDL15506 |
|  | EDL15507 |
|  | EDL15508 |
|  | D63334 |
|  | BAA09647 |
|  | AF128210 |
|  | AAF22551 |
|  | AF128211 |
|  | AAF22552 |
|  | AF128212 |
|  | AAF22553 |
|  | AF128213 |
|  | AAF22554 |
|  | AK153729 |
|  | AV363060 |
|  | BC116851 |
|  | AAI16852 |
|  | BC116855 |
|  | AAI16856 |
|  | M86672 |
|  | AAA39292 |
|  | P43431 |
|  | NC\_005101 |
|  | NM\_053390 |
|  | NP\_445842 |
|  | AC\_000070 |
|  | AABR07011640 |
|  | AABR07011641 |
|  | AAHX01017684 |
|  | AAHX01017685 |
|  | CH473976 |
|  | EDM00915 |
|  | EDM00916 |
|  | AF177031 |
|  | AAD51364 |
|  | Q9R103 |
|  | NC\_018914 |
|  | AMYH02008013 |
|  | AC\_000025 |
|  | AAHY01027630 |
|  | BC141596 |
|  | AAI41597 |
|  | BC146595 |
|  | AAI46596 |
|  | XM\_005247435 |
|  | XP\_005247492 |
|  | AC\_000135 |
|  | ABBA01058801 |
|  | S82412 |
|  | S82414 |
|  | S82417 |
|  | S82418 |
|  | S82419 |
|  | NT\_005612 |
|  | NW\_001838884 |
|  | NW\_921807 |
|  | AC\_000046 |
|  | Q7LDP2 |
|  | NT\_039240 |
|  | NW\_001030721 |
|  | Q549G3 |
|  | Q9QUT1 |
|  | NW\_047625 |
|  | NW\_001084805 |

---

|  |  |
| --- | --- |
| Swiss-Prot ID | IL12A\_MOUSE |
|  | IL12A\_RAT |
|  | IL12A\_HUMAN |
|  | E9QP58\_MOUSE |
|  | Q549G3\_MOUSE |
|  | O60595\_HUMAN |

---

|  |  |
| --- | --- |
| Cell Localization | Secreted |
|  | Extracellular |

---

|  |  |
| --- | --- |
| Ensembl ID | ENSG00000168811 |
|  | ENSP00000303231.2 |
|  | ENST00000305579.7 |
|  | ENSP00000419046.1 |
|  | ENST00000466512.1 |
|  | ENSP00000420184.1 |
|  | ENST00000480787.5 |
|  | ENSMUSG00000027776 |
|  | ENSMUSP00000029345.5 |
|  | ENSMUST00000029345.11 |
|  | ENSMUSP00000103446.2 |
|  | ENSMUST00000107816.3 |
|  | ENSRNOG00000009468 |
|  | ENSRNOP00000012832.1 |
|  | ENSRNOT00000012831.3 |
|  | ENSP00000303231 |
|  | ENST00000305579 |
|  | ENSMUSP00000029345 |
|  | ENSMUST00000029345 |
|  | ENSMUSP00000103446 |
|  | ENSMUST00000107816 |
|  | ENSRNOP00000012832 |
|  | ENSRNOT00000012831 |
|  | ENSP00000419046 |
|  | ENST00000466512 |

---

|  |  |
| --- | --- |
| MGI ID | MGI:96539 |
|  | 96539 |

---

|  |  |
| --- | --- |
| RGD ID | 620562 |

---

|  |  |
| --- | --- |
| Unigene ID | Hs.673 |
|  | Mm.103783 |
|  | Rn.207199 |

---

|  |  |
| --- | --- |
| Homologene ID | 681 |

---

|  |  |
| --- | --- |
| Shape | Rhomb |

---

|  |  |
| --- | --- |
| IPI ID | IPI00120355 |
|  | IPI00206535 |
|  | IPI00182737 |
|  | IPI00929795 |
|  | IPI00796173 |
|  | IPI00475347 |

---
